# miR-330 targeting *BCO2* is involved in carotenoid metabolism to regulate skin pigmentation in rainbow trout (*Oncorhynchus mykiss*)

**DOI:** 10.1186/s12864-023-09173-z

**Published:** 2023-03-16

**Authors:** Shenji Wu, Lu Zhao, Jinqiang Huang, Yongjuan Li, Zhe Liu, Dongqiang Zhang

**Affiliations:** 1 College of Animal Science and Technology, Gansu Agricultural University, Lanzhou, 730070 China; 2 College of Science, Gansu Agricultural University, Lanzhou, 730070 China

**Keywords:** MiR-330, *BCO2*, Rainbow trout, Skin pigmentation, Expression analysis, Functional analysis

## Abstract

**Background:**

MicroRNAs (miRNAs) play a critical role in regulating skin pigmentation. As a key economic trait, skin color directly affects the market value of rainbow trout (*Oncorhynchus mykiss*), however, the regulatory mechanism of most miRNAs in fish skin color is still unclear.

**Results:**

In this study, the full-length cDNA sequence of β-carotene oxygenase 2 (*BCO2*, a key regulator of carotenoid metabolism) from the rainbow trout was obtained using rapid-amplification of cDNA ends (RACE) technology, and qRT-PCR was used to investigate the differential expression of miR-330 and *BCO2* in 14 developmental stages and 13 tissues between wild-type rainbow trout (WTrt) and yellow mutant rainbow trout (YMrt). Additionally, the function of miR-330 was verified by overexpression and silencing in vitro and in vivo. The results showed that the complete cDNA sequence of *BCO2* was 2057 bp with a 1707 bp ORF, encoding a 568 amino acid protein having a molecular weight of 64.07 kD. Sequence alignment revealed that higher conservation of BCO2 protein amongst fishes than amongst other vertebrates, which was further confirmed by phylogenetic analysis. The analysis of spatial and temporal expression patterns suggested that *BCO2* and miR-330 were abundantly expressed from fertilized-stage to multi-cell as well as in the dorsal and ventral skin of WTrt and YMrt, and their expression patterns were opposite in most of the same periods and tissues. In vitro, luciferase reporter assay confirmed that *BCO2* was a direct target of miR-330, and transfection of miR-330 mimics into rainbow trout liver cells resulted in a decrease in the expression of *BCO2*; conversely, miR-330 inhibitor had the opposite effect to the miR-330 mimics. In vivo, miR-330 agomir significantly decreased *BCO2* expression in dorsal skin, tail fin, and liver. Furthermore, overexpression of miR-330 could suppress cell proliferation and induce apoptosis.

**Conclusion:**

Our results showed that miR-330 is involved in the regulation of skin pigmentation in rainbow trout by targeting *BCO2* and shows its promise as a potential molecular target to assist the selection of rainbow trout with better skin color patterns.

## Introduction

The coloration of fish is one of the most significant quality characteristics dictating the market acceptance and price for human consumption and ornamental use, and it is generally considered to depend on the type and number of chromatophores included in the epidermis and in the dermis [1]. In comparison with mammals, where only one pigment cell type is present, six types of pigment cells (i.e. melanophores, xanthophores, erythrophores, iridophores, leucophores, and cyanophores) have been identified in fish skin, which provides excellent materials for the study of biological development and evolutionary applications [2]. Extensive studies have illustrated that the formation of skin color pattern is controlled by a series of complex and well-balanced programs of gene activation and silencing [3]. Within this pigmentation system, there are multiple layers of molecular regulation, and the ability of microRNAs (miRNAs) to achieve sequence-specific regulation of gene function could obviously prove to be of major functional importance [4].

MiRNAs, defined as transcripts approximately 22 nucleotides in length and lacking protein-coding capacity, negatively regulate target genes through binding to the 3′-untranslated region (3'-UTR) of mRNAs in the transcriptional or post-transcriptional level [5]. In mammals, over 60% of mRNAs are thought to be regulated by miRNAs [6]. Since their initial discovery in the 1993, miRNAs have been successively proven to participate in various biological processes, including cell development, differentiation, cell proliferation, and apoptosis [7]. In recent years, miRNAs have also been suggested to perform a critical role in regulating skin pigmentation. For example, overexpression of miR-508-3p in Lama glama (*Alpacas*) caused a decrease in melanin production by downregulating microphtalmia-associated transcription factor (*MITF*) [8]. MiR-206 was reported to be involved in skin pigmentation of koi carp (*Cyprinus carpio* L.), and its silencing in vivo could suppress the transcription level of melanocortin 1 receptor (*MC1R*) and its downstream genes [9]. Similarly, in common carp, miR-429 downregulation in vivo led to a substantial change in skin melanin content via directly targeting forkhead box D3 (*FOXD3*) [10]. In our previous study, we studied the miRNA expression profiles of rainbow trout (*Oncorhynchus mykiss*) with black skin (wild-type rainbow trout, WTrt) vs. yellow skin (yellow mutant rainbow trout, YMrt), and found that miR-330 was expressed differentially; in addition, the results of target genes prediction analysis via three bioinformatics software showed that miR-330 binds to the 3'-UTR of β-carotene oxygenase 2 (*BCO2*), suggesting that miR-330 may be a key regulator of skin pigmentation in rainbow trout [11].

Yellow and red hues, given by the storage of carotenoids in skin or muscle, are important for commercial fish species, such as rainbow trout, koi carp, and Atlantic salmon (*Salmo salar*) [12]. *BCO2* encodes a carotenoid-cleavage enzyme that mediates the asymmetrical cleavage of carotenoids from the diet to prevent excessive deposition of carotenoids in the body, in other words, *BCO2* expression level is closely related to tissue-specific yellow phenotype [13]. Based on the results of our previous study, we found that *BCO2* plays an important role in the formation of rainbow trout yellow skin color [11, 14]. Accordingly, it is necessary to further verify whether miR-330 has a regulatory function on *BCO2*.

Rainbow trout, a key economic freshwater fish throughout the world, is widely accepted by consumers. As a mutant species, YMrt has the phenomenon of skin color variation in the long-term intensive farming process, which seriously restricts the healthy development of aquaculture. However, to date, researches on rainbow trout skin color have been limited to expression patterns analysis and pigment cells observation [15, 16], and the specific regulation mechanism of key skin color related genes or miRNAs are barely understood. As an essential regulator of yellow skin, the post-transcriptional regulatory mechanism of *BCO2* at the miRNA level was further investigated in this study. Firstly, the full-length cDNA sequence of *BCO2* was obtained by rapid-amplification of cDNA ends (RACE) technique, and expression patterns of miR-330 and *BCO2* in different developmental stages and tissues between WTrt and YMrt were characterized by quantitative real-time PCR (qRT-PCR). Then, we used luciferase reporter assay to study the regulatory effect between miR-330 and *BCO2*, and the effects of miR-330 on *BCO2* expression, cell proliferation, and apoptosis were observed by overexpression and inhibition of miR-330 in liver cells. Finally, we overexpressed miR-330 in vivo with agomir to further verify the function. The findings demonstrated the negative regulation of miR-330 on *BCO2*, which provide more information on the function of miRNAs in regulating fish skin color and suggest a molecular basis for breeding with skin color as the target trait.

## Results

### Sequence analysis of BCO2 cDNA

**Fig. 1:**
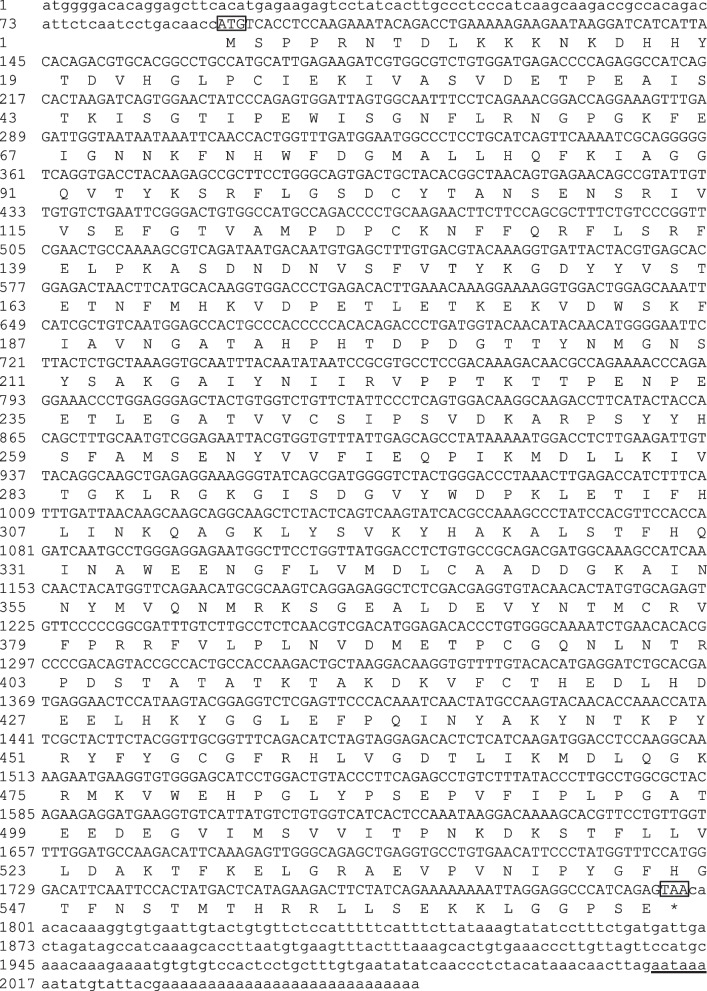
The full-length cDNA and deduced amino-acid sequences of β-carotene oxygenase 2 (*BCO2*) in rainbow trout. Boxes represent start codon (ATG) and stop codon (TAA). * Means no amino acid is encoded

**Fig. 2:**
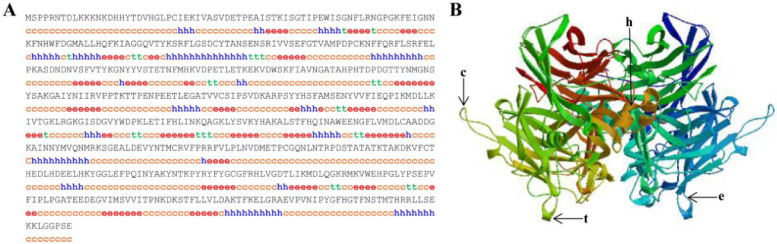
Predicted second and tertiary structures of BCO2 protein in rainbow trout. **A** Second structure. **B** Tertiary structure. c, random coils; e, extended strands; h, α-helices; t, β-turns

The full-length cDNA sequence of *BCO2* (GenBank accession number: OQ053250) was assembled as a 2057 bp and contained a 1707 bp open reading frame (ORF), which contained a 91 bp 5′-UTR and a 259 bp 3′-UTR, encoding a total of 568 amino acids and polyadenylation signal sequence (AATAAA) was found in the 3′-UTR (Fig. 1). Protparam analysis showed BCO2 corresponds to a molecular mass of 64.07 kD (molecular formula: C_2875_H_4417_N_763_O_850_S_25_), with a theoretical pI of 6.84 and grand average of hydropathicity of –0.476, suggesting that BCO2 is a hydrophilic protein. Protscale analysis showed the highest hydrophilicity (hydrophobic parameter: –3.367) was lysine (K) at position 14, and the highest hydrophobicity (hydrophobic parameter: 2.400) was serine (S) at position 507. Further bioinformatical analyses indicated that the RPE65 domain was found in polypeptide of BCO2 (residues 31–550), which belonged to a non-transmembrane protein. The predicted secondary structure revealed that BCO2 included random coils (57.04%), extended strands (21.30%), α-helices (17.08%), and β-turns (4.58%) (Fig. 2A). From tertiary structure, it can be clearly seen that BCO2 protein was also mainly composed of random coils and extended strands (Fig. 2B).

### Comparison of BCO2 proteins and phylogenetic tree construction

**Fig. 3:**
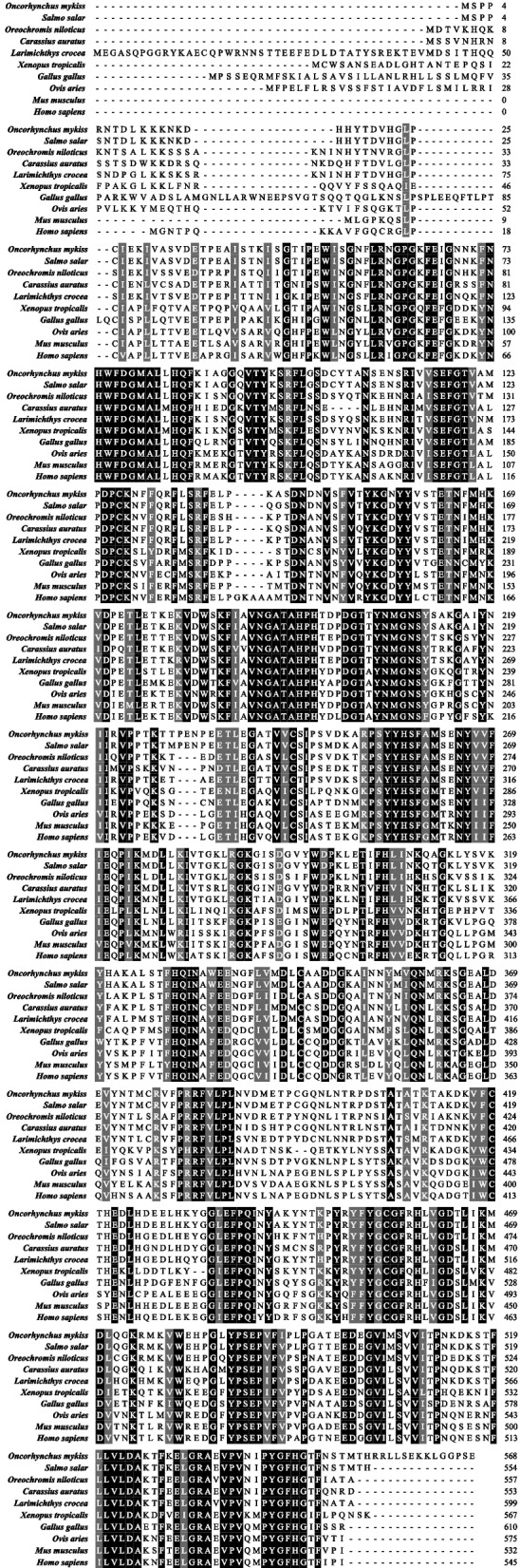
Multiple alignments of BCO2 amino acid sequences in rainbow trout with those of other vertebrates. Grey represents amino acid sequences with high similarity. Black indicates the same amino acid sequences

To analyze the similarity of amino acid sequences of BCO2 protein among different species, we compared the amino acid sequences of rainbow trout BCO2 with those of other vertebrates BCO2. The results showed that the deduced amino acid sequence of BCO2 in rainbow trout shared the highest homology with *Salmo salar* BCO2 (98.01%, NP_001266006.1), and there is 76.52–79.70% homology with BCO2 of *Carassius auratus* (XP_026138906.1), *Oreochromis niloticus* (XP_013131987.2), and *Larimichthys crocea* (XP_010727865.2), 54.17–61.48% homology with BCO2 of *Xenopus tropicalis* (XP_031760855.1), *Homo sapiens* (NP_001032367.3), *Ovis aries* (NP_001152750.2), *Mus musculus* (NP_573480.1), and *Gallus gallus* (XP_004948199.2) (Fig. 3).

**Fig. 4:**
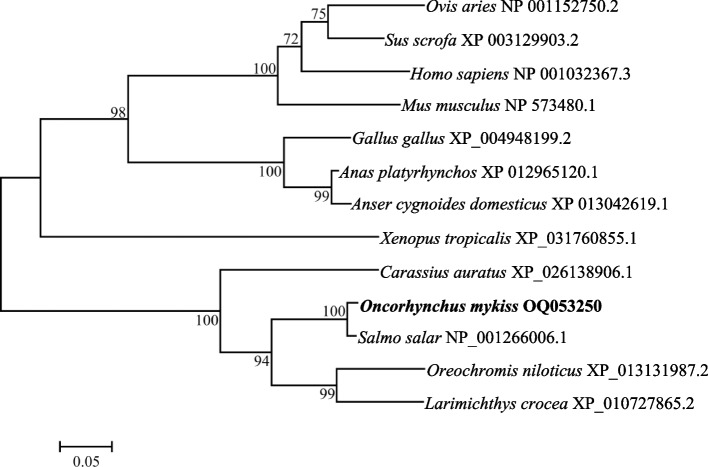
Phylogenetic tree generated using maximum likelihood algorithm from the BCO2 amino acid sequences of rainbow trout and other vertebrates. Numbers at the nodes indicate bootstrap values. Scale bar below the tree measures evolutionary distances in substitutions per site

To evaluate the molecular evolutionary relationships of BCO2, a phylogenetic tree based on the protein sequences was constructed by maximum likelihood algorithm (Fig. 4). In general, species from the same taxonomic status were clustered together and formed a separated subgroup. As shown in the result, BCO2 of rainbow trout is clustered first with BCO2 from *Salmo salar*, then formed a sister group with those of other fishes, and further with those of amphibian, poultry, and mammals.

### Expression patterns analysis of BCO2 and miR-330 in different developmental stages and tissues between WTrt and YMrt

**Fig. 5:**
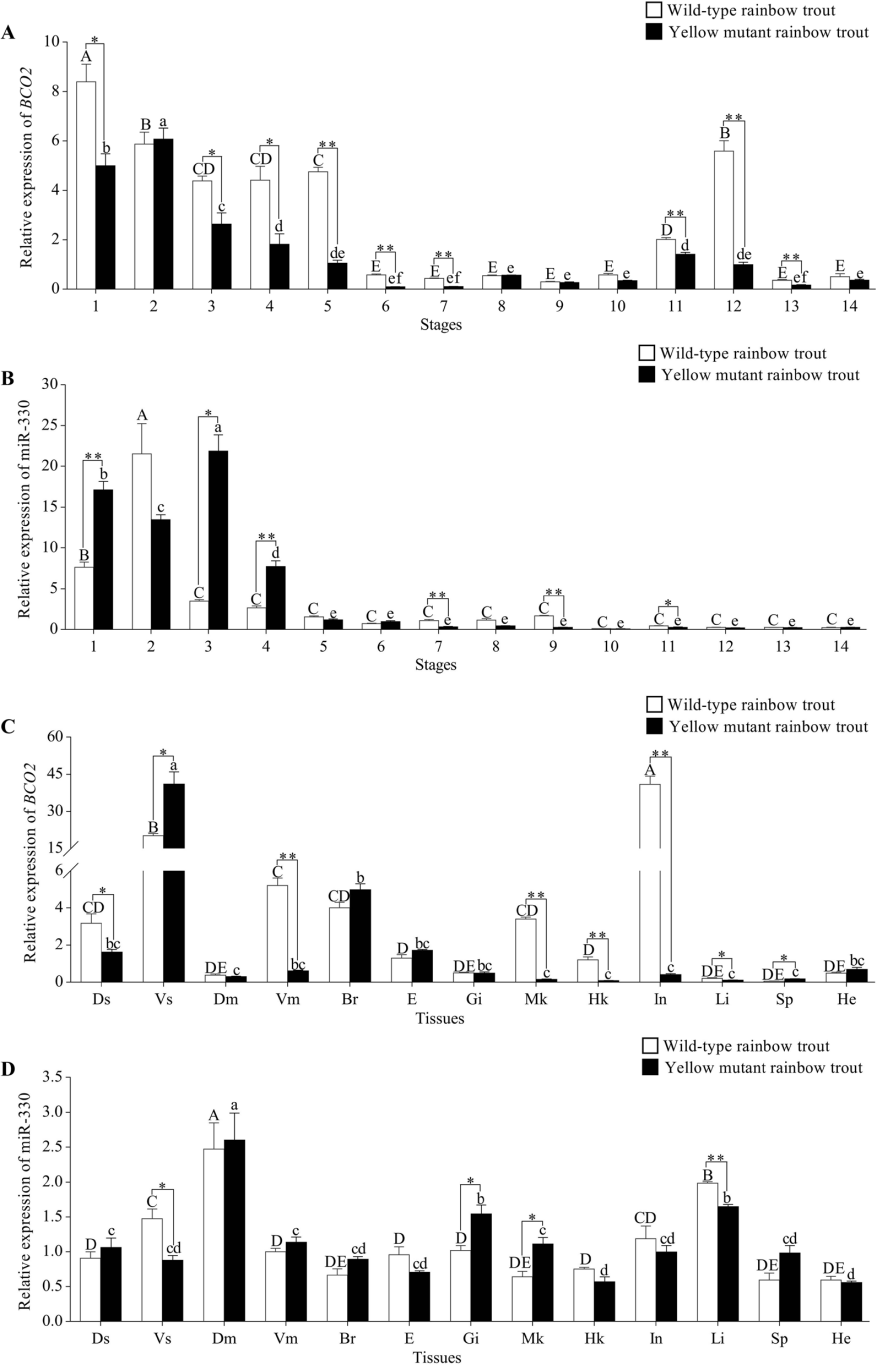
Expression profiles of *BCO2* and miR-330 in wild-type rainbow trout (WTrt) and yellow mutant rainbow trout (YMrt). **A** and **B** Expression patterns of *BCO2* and miR-330 at different developmental stages. (**C** and **D**) Expression patterns of *BCO2* and miR-330 in different tissues. 1: Fertilized-stage; 2: 4-cell; 3: 16-cell; 4: Multi-cell; 5: Blastula; 6: Gastrula; 7: Neurula; 8: Somites; 9: Heartbeating; 10: 1 day post hatching (1 dph); 11: 10 dph; 12: 1 month post hatching (1 mph); 13: 2 mph; 14: 3 mph. Ds: Dorsal skin; Vs: Ventral skin; Dm: Dorsal muscle; Vm: Ventral muscle; Br: Brain; E: Eye; Gi: Gill; Mk: Midkidney; Hk: Headkidney; In: Intestine; Li: Liver; Sp: Spleen; He: Heart. Reference gene: *β-actin* and *U6*; n = 3 different independent samples. Capital alphabet superscripts were used to indicate the differences within WTrt, and lowercase alphabet superscripts were used to indicate the differences within YMrt, different letters represent significant difference (*P* < 0.05) (one-way ANOVA). The difference analysis in the same developmental stage or tissue between WTrt and YMrt is indicated by * and **, *: *P* < 0.05, **: *P* < 0.01 (student’s t-test)

To gain insight into the role of *BCO2* and *miR-330* in skin pigmentation of WTrt and YMrt, it is essential to have precise information on their temporal and spatial expression patterns. In 14 different development stages, the results indicated that the expression of both *BCO2* and *miR-330* were mainly concentrated in fertilized-stage to multi-cell, and their expression patterns were opposite in these four stages (Fig. 5A and B). In addition, except for 4-cell and somites, the expression level of *BCO2* was higher in WTrt than in YMrt in other same stages, and most of them have significant differences (*P* < 0.05), including fertilized-stage, 16-cell, multi-cell, blastula, gastrula, neurula, 10 days post hatching (10 dph), 1 month post hatching (1 mph), and 2 mph. In 13 various tissues, the highest expression level of *BCO2* in WTrt and YMrt were detected in the intestine and ventral skin, respectively, whose expression was significantly higher than other tissues (*P* < 0.05) (Fig. 5C). For miR-330, a higher expression level was found in the dorsal muscle and liver of WTrt and YMrt, and relative expression in other tissues were comparable (Fig. 5D). Moreover, the expression patterns of *BCO2* and miR-330 in dorsal skin, ventral skin, dorsal muscle, ventral muscle, eye, gill, midkidney, and heart were opposite between WTrt and YMrt. Given those results, we speculated that miR-330 is a potential regulator of the pigmentation process in rainbow trout.

### MiR-330 directly targeted BCO2

**Fig. 6:**
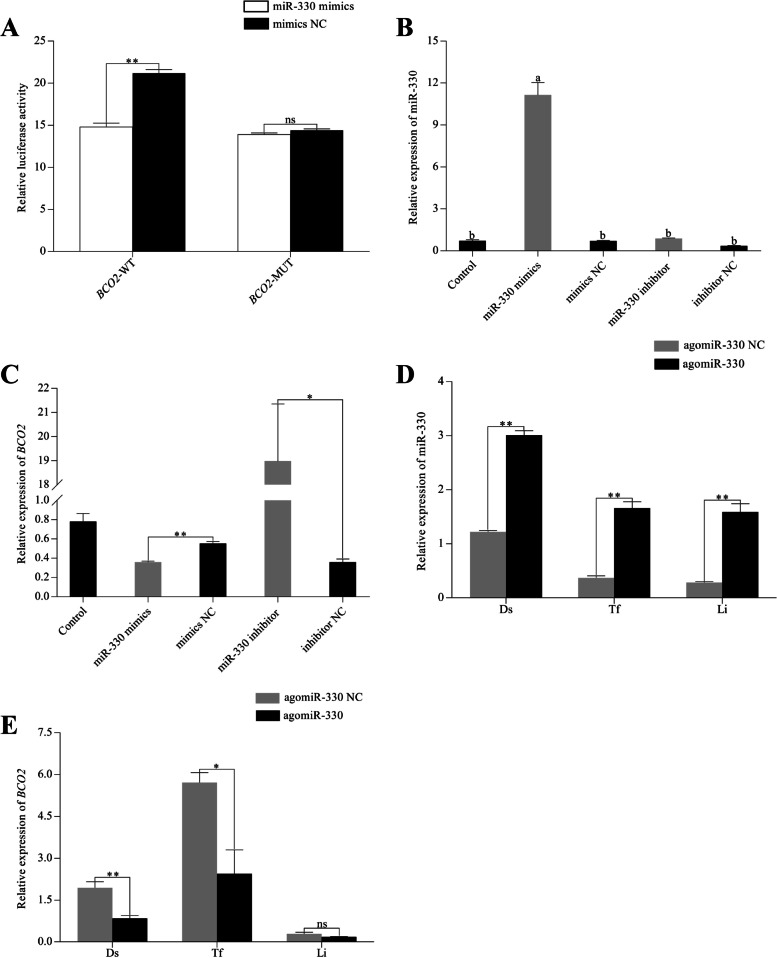
MiR-330 negatively regulated *BCO2* expression. **A** Dual-luciferase analysis of miR-330 mimics/mimics NC co-transfected with either *BCO2*-WT or *BCO2*-MUT into HEK293T cells. **B** and **C** The expression of miR-330 and *BCO2* in rainbow trout liver cells after transfection with miR-330 mimics and miR-330 inhibitor. **D** and **E** The effect of miR-330 agomir on the expression of miR-330 and *BCO2* in dorsal skin (Ds), tail fin (Tf), and liver (Li). The different lowercase letters above the bars represent significant differences (*P* < 0.05); *: *P* < 0.05, **: *P* < 0.01, ns:* P* > 0.05

To reveal the potential mechanism of miR-330 during skin pigmentation in rainbow trout, several online analysis tools were used to predict the targets of miR-330 and found that a potential binding site were located from 1971 to 1977 nt (5′…TGCTTTG…3′) in the 3′-UTR region of *BCO2*, which was matched miR-330 seed sequence (5′…CAAAGCA…3′). As shown in Fig. 6A, luciferase reporter assay showed that the luciferase activity of HEK293T cells was significantly reduced (*P* < 0.01) after co-transfection with miR-330 mimics and *BCO2*-wild-type receptor vectors (*BCO2*-WT) compared with mimics negative control (NC), while the luciferase activity of HEK293T cells that were transfected with *BCO2*-mutant receptor vectors (*BCO2*-MUT) was not affected by miR-330 mimics, suggesting *BCO2* is a direct target of miR-330.

### MiR-330 negatively regulated BCO2 expression in liver cells

To further explore the underlying regulation mechanism of miR-330 on *BCO2*, we treated liver cells with miR-330 mimics, miR-330 inhibitor, and their corresponding NC. According to the qRT-PCR results, the relative expression of miR-330 in mimics group reached nearly 15 times in contrast with other groups, while *BCO2* expression level was significantly reduced (*P* < 0.01) (Fig. 6B and C). Conversely, miR-330 inhibitor resulted in contradictory outcomes (*P* < 0.05) compared with inhibitor NC group (Fig. 6C). The results suggested that miR-330 and *BCO2* were negatively correlated.

### MiR-330 restrained liver cells proliferation

**Fig.7:**
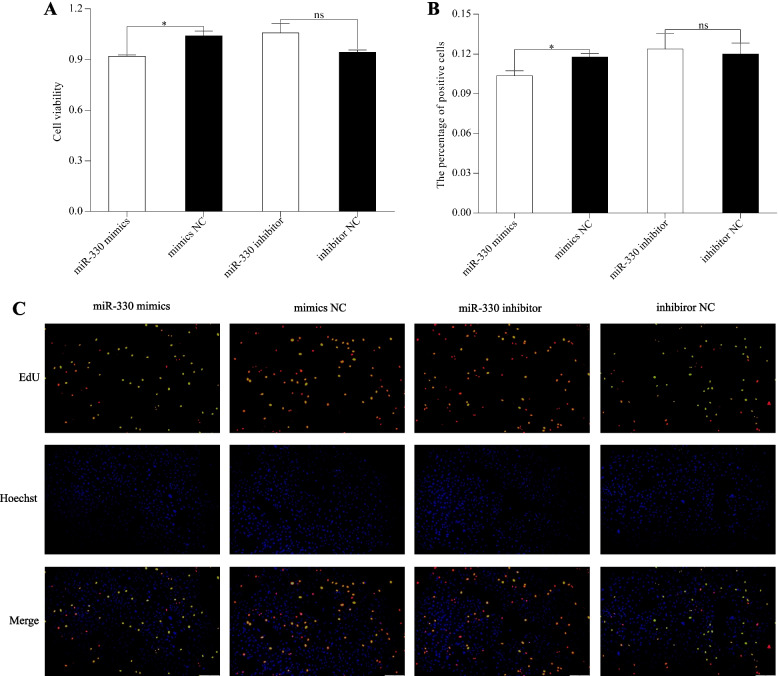
The effect of miR-330 on cell viability and proliferation of rainbow trout liver cells. **A** The viability of liver cells using CCK-8 assay after miR-330 mimics and miR-330 inhibitor were transfected into liver cells. **B** and** C** Percentage of EdU-positive cells of liver cells (red and yellow) after miR-330 mimics and miR-330 inhibitor treatment by EdU staining (20 ×). *: *P* < 0.05, ns: *P* > 0.05

Having confirmed the interaction of miR-330 and *BCO2*, we next sought to explore the biological role of miR-330 in liver cells. CCK-8 assay revealed that the viability of liver cells declined remarkably in miR-330 mimics group (*P* < 0.05), whereas the viability enhanced in miR-330 inhibitor group (*P* > 0.05) (Fig. 7A). Meanwhile, cell proliferation was estimated through EdU staining, and the results were consistent with CCK-8 assay (Fig. 7B and C). In other words, miR-330 substantially suppressed the proliferation of liver cells.

### MiR-330 induced liver cells apoptosis

**Fig. 8:**
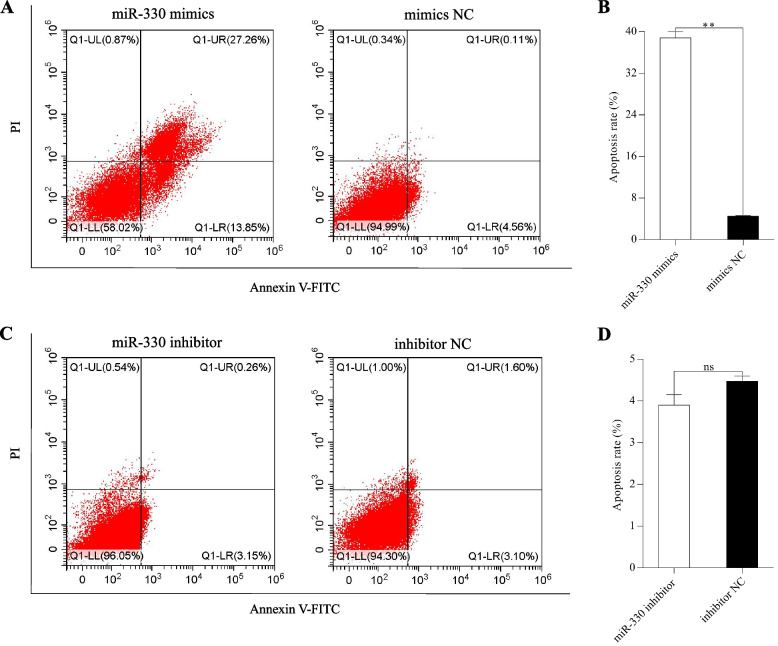
Rainbow trout liver cells apoptosis was controlled by the change of miR-330 expression. **A** and** B** Cell apoptosis rate of liver cells after overexpression of miR-330. **C **and** D** Cell apoptosis rate of liver cells after inhibition of miR-330. Cell apoptosis rate (%) indicates the sum of early and late apoptotic cells ratio (Q1-LR + Q1-UR). **: *P* < 0.01, ns: *P* > 0.05

To investigate whether the anti-proliferative activity of miR-330 was correlated with an apoptotic effect, flow cytometry was then carried out to detect the impact of miR-330 on the number of apoptotic cells in each group. As shown in Fig. 8A–D, the apoptosis rate of liver cells rose dramatically in miR-330 mimics group (*P* < 0.01), but declined in miR-330 inhibitor group (*P* > 0.05), indicating that miR-330 had a positive effect on liver cells apoptosis.

### Effect of miR-330 agomir on the expression of BCO2 in vivo

After explaining the regulatory relationship between miR-330 and *BCO2 *in vitro, we further verified the regulatory relationship between miR-330 and *BCO2 *in vivo by agomir. As expected, the results showed that the expression level of *BCO2* was down-regulated to varying degrees in dorsal skin, tail fin, and liver of fish with a significant increase in the expression of miR-330 after treated with agomiR-330 (Fig. 6D and E).

## Discussion

Rainbow trout, an important cold-water economic fish, is becoming increasingly popular for aquaculture production in China in recent years. Skin color is a key economic trait for this species, as it has a strong impact on quality and market value when rainbow trout are marketed. In addition to the direct influence of genes, other regulators, such as miRNAs, are reported to play crucial roles in fish skin color via regulating the expression of key genes in specific pigment synthesis pathways [9, 10]. In our previous study, although 275 differentially expressed miRNAs were identified in skin between WTrt and YMrt, the specific regulatory mechanism of miRNAs underlying rainbow trout skin pigmentation has not yet been elucidated. The aim of the current study was to investigate the regulatory function of miR-330 on *BCO2* by in vitro and in vivo experiments. Understanding the effects of miRNAs on regulating rainbow trout skin color can provide a molecular basis for breeding with skin color as the target trait.

Chromatophores of fish emanate from neural crest cells during embryonic development, and then differentiate into various pigment cell types under the action of cell type specific genes expression [17, 18]. Thus, analysis of expression patterns of key genes at different developmental stages is essential to understand the skin pigmentation. Unlike most genes, the way of *BCO2* involving in skin color regulation is to catalyze the oxidative cleavage of yellow carotenoids acquired from the diet into colorless apocarotenoids; besides, the expression level of *BCO2* is negatively corrected with yellow phenotype [19, 20]. Several studies have shown that mutations in *BCO2* gene had significant impact on the carotenoid metabolism, resulting in visible increase in yellow pigment production of skin in chicken (*Gallus gallus domesticus*) and reptiles [21, 22]. Similarly, loss-of-function mutations in *BCO2* in sheep (*Ovis aries*), cows (*Bovine*), and rabbits (*Leporidae*) caused a yellow fat phenotype [23–25]. In East African cichlid fish (*Tropheus duboisi*), Ahi et al. revealed that *BCO2* expression in white skin was obvious higher than that in yellow skin [20]. Consistent with these researches, our data showed that similar effects of *BCO2* on carotenoid-based skin color polymorphism exist in rainbow trout. In addition, miR-330 and *BCO2* were highly expressed from fertilized-stage to multi-cell in WTrt and YMrt. Given that *BCO2* is required for normal embryogenesis in mammals through maintaining vitamin A balance in vivo [26], we hypothesize that both of them may play important roles in embryogenesis of rainbow trout. *BCO2* showed relative higher expression levels in the WTrt and YMrt dorsal skin at developmental stages of 10 dph and 1 mph; however, miR-330 showed almost no expression in the same time frames. The results may indicate that the basal expression level of miR-330 can exert a regulatory effect on *BCO2*, and also reflect the complexity of the molecular regulation mechanism of skin color formation in rainbow trout. Furthermore, previous studies found that overexpression of miR-330 can induce a significant reduction in the production of melanin in human and mouse melanocytes by targeting tyrosinase (*TYR*) gene, encoding a key rate-limiting enzyme in melanogenesis [27, 28], suggesting lower expression of miR-330 after the gastrula stage may contribute to the generation of dark skin in WTrt.

Interestingly, widespread expression of miR-330 and *BCO2* was also found in all tissues tested. From the expression patterns of dorsal and ventral skin between WTrt and YMrt, it is again demonstrated that the low expression of *BCO2* is conductive to the carotenoid accumulation in skin. In the process of carotenoid metabolism, the intestine is a major site for absorption and bioconversion [29]. In chicken, lower expression level of *BCO2* was detected in yellow skin, but not in the intestine [25]. Our data possibly indicated *BCO2* expression level in the intestine is tightly associated with the difference of skin carotenoid deposition between WTrt and YMrt. In addition to the skin, muscle also contains relatively higher contents of carotenoids [30]. As a mitochondrial protein, *BCO2* is responsible for changes in carotenoid accumulation in the muscle of Chinook salmon (*Oncorhynchus tshawytscha*) [31]. In our study, we observed opposite expression patterns of miR-330 and *BCO2* in the dorsal and ventral muscle of WTrt and YMrt, which implied a key role of miR-330 in regulating flesh color.

It’s was widely accepted that miRNAs suppress targets by binding their 3'-UTR to induce protein translational inhibition or mRNA degradation [32]. To identify whether miR-330 could target *BCO2* gene, we examined the changes in *BCO2* expression in rainbow trout liver cells after overexpression or inhibition of miR-330. Notably, *BCO2* expression was apparently inhibited and promoted by miR-330 mimics and inhibitor when compared with the control. Agomir, a cholesterol-conjugated double-stranded RNA molecule, has been widely used in several fish species to verify the relationship between miRNAs and their target genes in vivo [33–35], which was also adopted to further confirm the effect of miR-330 overexpression on *BCO2*. As expected, we found that miR-330 expression level was significantly enhanced in the dorsal skin, tail fin and liver, while the *BCO2* gene was marked downregulation. Additionally, luciferase reporter assay verified that miR-330 inhibited *BCO2* expression by specifically binding its 3'-UTR. All of these experiments collectively supported that miR-330 was involved in carotenoid accumulation to regulate skin pigmentation via targeting *BCO2*.

Cell proliferation and apoptosis are normal physiological phenomena that help to maintain the number of cells in the body during the development, which are implicated with the process of skin pigmentation [36]. Previous studies have reported that melanophores proliferation and apoptosis are regulated by numerous key melanin-related genes, such as *MITF*, Kit type III receptor tyrosine kinase (*KIT*), and G protein subunit alpha i2 (*GNAI2*), thereby further affecting melanin synthesis [37–39]. Although the biologic functions of most miRNAs are not yet clarified, there is growing evidence that they perform essential roles in cell proliferation and apoptosis [7]. So far, several miRNAs, such as miR-330, miR-7013, and miR-18a, have been identified to regulate the apoptotic pathways of melanoma cells via targeting specific genes [40–42]. Moreover, overexpression of miR-330 can also suppress proliferation and induce apoptosis in other cell types [43, 44], thus, miR-330 is often considered a pro-apoptotic factor. As its target, *BCO2* gene was also found to involve in apoptosis activity. A study in zebrafish (*Danio rerio*) revealed that *BCO2* deficiency promoted blood cell apoptosis at larval stages [45]. In this study, our results showed that overexpression of miR-330 accelerated cellular apoptosis, while the opposite trend was shown in proliferation. Therefore, miR-330 is a positive regulator of apoptosis in rainbow trout liver cells. However, the effect of miR-330 on xanthophores proliferation and apoptosis remains to be further studied.

## Conclusion

In the present study, we investigated the regulatory mechanism of miR-330 in rainbow trout skin pigmentation in vitro and in vivo. The results revealed that the full-length cDNA of *BCO2* gene was 2057 bp encoding 568 amino acids, which was shown to be a target of miR-330, and both of them were highly expressed from fertilized-stage to multi-cell as well as in the dorsal and ventral skin of WTrt and YMrt. Treatment with miR-330 mimics or agomir resulted in a significantly decrease of *BCO2* expression, and the opposite result was obtained after processing with miR-330 inhibitor. Additionally, overexpression of miR-330 could obviously suppress rainbow trout liver cells proliferation and induce apoptosis. The research here confirmed a functional role of miR-330 in regulating skin pigmentation of rainbow trout via targeting *BCO2* and shows its promise as a potential molecular target to assist the selection of rainbow trout with better skin color patterns.

## Materials and methods

### Embryo and tissue collection

Eggs of WTrt and YMrt from full-sib families were obtained from, and raised at the Aquatic Science Training Center of Gansu Agricultural University in Gansu province, China. During the incubation phase, all fertilized eggs were kept in a cylindrical plastics water tank with temperature 12 ± 0.5 °C, pH = 7–8, and dissolved oxygen (DO) = 9 ± 0.5 mg/L. After hatching, the fish were maintained in a 18 ± 0.5 °C flow-through water system (pH = 7–8, DO = 9 ± 0.5 mg/L). Embryos and larvae were collected at the following stages as described previously [16]: fertilized-stage, 4-cell, 16-cell, multi-cell, blastula, gastrula, neurula, somites, heartbeating, 1 dph, 10 dph, 1 mph, 2 mph, and 3 mph (12 embryos or six larvae (from 1 dph to 1 mph) or one fish dorsal skin (2 mph and 3 mph) were collected as one sample, n = 3). Samples were also obtained from 12 mph WTrt and YMrt after anaesthetization by MS-222 (Sigma Aldrich Co., St. Louis, USA). Individual tissues including dorsal skin, ventral skin, dorsal muscle, ventral muscle, brain, eye, gill, midkidney, headkidney, intestine, liver, spleen, and heart were quickly collected and immediately stored in liquid nitrogen until RNA extraction. Three independent individuals were used for replication. All the experimental procedures were carried out in accordance with the Guidelines for the Care and Use of Laboratory Animals in China, and the protocol was approved by the Animal Experimentation Ethics Committee at Gansu Agricultural University, China (GSAU-Eth-AST-2021–004).

### Molecular cloning of full-length cDNA of BCO2

Based on the skin transcriptome analysis of rainbow trout [11], partial cDNA sequence of *BCO2* was obtained. Gene specific primers were designed to amplify the full-length cDNA of *BCO2* (Table 1). The first strand cDNA for RACE was prepared using the SMARTer RACE 5′/3′ Kit (Clontech, Mountain View, CA, USA). The PCR reaction system and condition of touchdown PCR (outer and UPM_long_) and nested PCR (inner and UPM_short_) for 5′RACE/3′RACE were conducted according to the manufacturer's instructions, respectively. Products from touchdown PCR or nested PCR were extracted and purified by Zymoclean™ Gel DNA Recovery Kit (ZYMO Research, USA). Purified RACE products were then ligated into linearized pRACE vector (a SMARTer RACE 5′/3′ Kit component) and transformed into stellar competent cells (Clontech, Mountain View, CA, USA). Positive colonies containing insert fragments of the expected size were identified by colony PCR, and three independent positive colonies were finally sequenced at Sangon (Shanghai, China).

**Table 1:** Primers information used in the study

Primers	Sequence (5' to 3')	Application
*BCO2*-F	CGTGGTGTTTATTGAGCAGCC	qRT-PCR
*BCO2*-R	GGCTTTGGCGTGATACTTGACT	qRT-PCR
*β-actin*-F	TGGGGCAGTATGGCTTGTATG	qRT-PCR
*β-actin*-R	CTCTGGCACCCTAATCACCTCT	qRT-PCR
*BCO2*-5′outer	GATTACGCCAAGCTTGGTTTTCTGGCGTTGTCTTTGTCGGA	5′RACE
*BCO2*-3′outer	GATTACGCCAAGCTTCGCTACTTCTACGGTTGCGGTTTCAG	3′RACE
*BCO2*-3′inter	GATTACGCCAAGCTTAAGGTGTGGGAGCATCCTGGACTGTA	3′RACE
UPM_long_	CTAATACGACTCACTATAGGGCAAGCAGTGGTATCAACGCAGAGT	5′RACE/3′RACE
UPM_short_	CTAATACGACTCACTATAGGGC	5′RACE/3′RACE
MiR-330	TTTATTAGCAAAGCACAGGGCCTGC	qRT-PCR
*U6*-F	GCTTCGGCAGCACATATACTAAAAT	qRT-PCR
*U6*-R	CGCTTCACGAATTTGCGTGTCAT	qRT-PCR
MiR-330 mimics	GCAAAGCACAGGGCCUGCAGAGA	Overexpression in vitro
	UCUGCAGGCCCUGUGCUUUGCUU	
mimics NC	UUCUCCGAACGUGUCACGUTT	Control of miR-330 mimics
	ACGUGACACGUUCGGAGAATT	
MiR-330 inhibitor	UCUCUGCAGGCCCUGUGCUUUGC	Downexpression in vitro
inhibitor NC	CAGUACUUUUGUGUAGUACAA	Control of miR-330 inhibitor
agomiR-330	GCAAAGCACAGGGCCUGCAGAGA	Overexpression in vivo
	UCUGCAGGCCCUGUGCUUUGCUU	
agomiR-330 NC	UUCUCCGAACGUGUCACGUTT	Control of agomiR-330
	ACGUGACACGUUCGGAGAATT	

### Bioinformatic analysis of BCO2

DNAMAN 9.0 software was used to splice the sequencing results to obtain the full-length sequence of *BCO2*. The results of the sequence were then blasted against NCBI non-redundant (nr) protein database by BLAST (www.ncbi.nlm.nih.gov/blast), and extract protein names for comparative analysis of gene and protein sequences. The online software Protparam (https://web.expasy.org/protparam) and Protscale (https://web.expasy.org/ protscale/) were used to analyze the basic physicochemical properties. Transmembrane structure and domains of the encoded protein were predicted using TMHMM (http://www.cbs.dtu.dk/services/TMHMM/) and SMART (http://smart.embl -heidelberg.de), respectively. SOPMA (https://npsa-prabi.ibcp.fr/) was used to analyze the protein secondary structure, and the protein tertiary structure was modeled using SWISS-MODEL (https://swissmodel.expasy.org/). The ClustalX 1.83 software was used to perform multiple alignments of amino acid sequences, and phylogenetic tree was constructed using the maximum likelihood algorithm with 1000 bootstrap replicates within the MEGA7.0 program [46].

### Dual-luciferase reporter assay

MiR-330 target genes were predicted using the RNAhybrid (v2.1.2) + svm_light (v6.01), Miranda (Version 3.3a), and TargetScan (Version 7.0). The *BCO2*-3′-UTR vectors were constructed by GENEWIZ (Suzhou, China). HEK293T cells were grown to 70–80% confluence in 24-well plate and co-transfected *BCO2*-WT/*BCO2*-MUT with miR-330 mimics or NC using INVI DNA RNA transfection Reagent (Invigentech, USA) (n = 3 different independent samples). The luciferase activity was measured 48 h post-transfection using Dual-Glo® Luciferase Assay System (Promega, USA) following the instruction of manufacturer. The 3′-UTR activity on the luciferase reporter gene was calculated as a ratio of firefly luciferase to renilla luciferase luminescence.

### Cell transfection

In our previous experiments, we found that miR-330 and *BCO2* were highly expressed in rainbow trout liver cells, thus, liver cells were used for functional analysis of miR-330 in vitro. MiR-330 mimics, mimics NC, miR-330 inhibitor, and inhibitor NC were all synthesized by Genepharma Co. Ltd (Shanhai, China). After rainbow trout liver cells reached 80% confluence in 24-well plate (n = 3 different independent samples), miR-330 mimics, miR-330 inhibitor, and their NC were transfected into cells using NVI DNA RNA transfection Reagent (Invigentech, USA) according to the manufacturer's protocol, respectively. Afterward, cells were incubated for 48 h and harvested for further analysis.

### Cell viability assay

After liver cells were transiently transfected with miR-330 mimics, miR-330 inhibitor, and their NC for 48 h in 96-well plate, the cell counting kit-8 (CCK-8, Solarbio, China) was introduced to detect cell viability (n = 3 different independent samples) [47]. 10 μL CCK-8 reagent was added into each well, and the cells were incubated at 20℃ for another 2 h. Following that, the optical density values were measured with a microplate reader (Thermo, UAS) at an absorbance of 450 nm.

### Cell proliferation assay

Cell proliferation was detected with 5-Ethynyl-2′-Deoxyuridine (EdU) assay. The EdU assay was performed according to the protocol of the BeyoClick™ EdU Cell Proliferation Kit with Alexa Fluor 555 (Beyotime, China). Briefly, normal growing liver cells were seed in a 24-well plate and treated with miR-330 mimics, miR-330 inhibitor, and their NC for 48 h (n = 3 different independent samples). Subsequently, the cells in each well were incubated with 500 μL of EdU (20 μM) for 24 h at 20℃. After being fixed with cell fixative (namely PBS containing 4% paraformaldehyde), the cells were exposed to 100 μL of click reaction buffer, followed by incubation at room temperature in dark for 30 min. Finally, 1 × Hoechst 33,342 was added to stain cell nuclei, and the positive cells were captured using a fluorescence microscope (Olympus IX71, Japan).

### Apoptosis assay

For cell apoptosis assay, propidium iodide (PI) (Beyotime, China) and annexin V-FITC (Beyotime, China) were used to detect cell apoptosis [48]. After miR-330 mimics, miR-330 inhibitor, and their NC transfection for 48 h, liver cells were washed with 1 mL annexin V-FITC binding buffer and stained by 5 μL annexin V-FITC and 5 μL propidium iodide (PI) for 10−15 min at 4 °C in the dark. Afterward, flow cytometry (Beckman, USA) was used to analyze cell apoptosis rate of each treatment.

### MiR-330 agomir injection in vivo

To determine the function of miR-330 in vivo, eight YMrt (25 ± 0.5 g) belonged to a full-sib family were selected and randomly divided into two groups: agomiR group and agomiR NC group. Four independent individuals were used for replication in each group. agomiR-330 and agomiR-330 NC were synthesized by Genepharma Co. Ltd (Shanhai, China). The animals in agomiR group and its negative control group were injected via tail vein with agomiR-330 and agomiR-330 NC for three consecutive days [33, 34]. On day four, the dorsal skin, tail fin, and liver samples were promptly removed and stored at − 80 °C for further assay.

### qRT-PCR analysis

Total RNA from different tissues and liver cells was isolated with Trizol reagent kit (Invitrogen, Carlsbad, CA, USA), and the cDNA was synthesized using a Mir-X miRNA First-Strand Synthesis Kit (Clontech, Mountain View, CA, USA) and a PrimerScript RT Reagent Kit with gDNA Eraser (Takara, Dalian, China). qRT-PCR was performed on LightCycler® 480 II Instrument (Roche, Basel, Switzerland) with SYBR Premix Ex Taq (Takara, Dalian, China). For *BCO2* quantification, the 20 μL reaction volume contained 10 μL of SYBR Premix Ex Taq II (2 ×), 1 μL of each sense and antisense primer (10 μM), 0.5 μL of cDNA, 7.5 μL of ddH_2_O. For miR-330 quantification, the 20 μL reaction volume contained 10 μL of SYBR Premix Ex Taq II (2 ×), 0.4 μL of each sense and antisense primer (10 μM), 1.6 μL of cDNA, 7.6 μL of ddH_2_O. PCR amplification procedure for all experiments were carried out at 95 ℃ for 30 s, followed by 40 cycles at 95 ℃ for 5 s and 60 ℃ for 30 s. Primers for qRT-PCR were designed by Primer v5.0 as listed in Table 1, and expression levels for *U6* and *β-actin* were used as the internal references to normalize the expression levels of miR-330 and *BCO2*, respectively [49]. Software SPSS version 22.0 (IBM Corp, Armonk, NY, USA) was used to conduct all statistical analyses and the results were presented as mean ± SD (Standard Deviation). Student’s t-test was conducted to compare the difference of the same stage or tissue between WTrt and YMrt, and one-way ANOVA was applied to compare the difference of various developmental stages or tissues in WTrt and YMrt. Statistically, *P* < 0.05 and *P* < 0.01 were considered significant and extremely significant differences, respectively.

## Data Availability

All data generated or analyzed during this study are included in this article, and the raw data can be obtained by contacting the corresponding author. The full-length cDNA sequence of *BCO2* has been submitted to National Center for Biotechnology Information (NCBI) (GenBank accession number: OQ053250).

## Abbreviations

WTrt: Wild-type rainbow trout
YMrt: Yellow mutant rainbow trout
ORF: Open reading frame
UTR: Untranslated region
qRT-PCR: Quantitative real-time PCR
NC: Negative control
*BCO2*-WT: *BCO2-*wild-type receptor vectors
*BCO2*-MUT: *BCO2-* mutant receptor vectors
MS-222: Tricaine methanesulfonate
1 dph: 1 Day post hatching
1 mph: 1 Month post hatching
UPM: Universal primer mix
